# Autophagy, Metabolism, and Alcohol-Related Liver Disease: Novel Modulators and Functions

**DOI:** 10.3390/ijms20205029

**Published:** 2019-10-11

**Authors:** Shengmin Yan, Bilon Khambu, Honghai Hong, Gang Liu, Nazmul Huda, Xiao-Ming Yin

**Affiliations:** Department of Pathology and Laboratory Medicine, Indiana University School of Medicine, Indianapolis, IN 46202, USA; bikhambu@iupui.edu (B.K.); honghong@iu.edu (H.H.); gangliu@iu.edu (G.L.); nhuda@iu.edu (N.H.)

**Keywords:** autophagy, alcohol-related liver disease, nuclear receptor, ncRNA, non-parenchymal cell

## Abstract

Alcohol-related liver disease (ALD) is caused by over-consumption of alcohol. ALD can develop a spectrum of pathological changes in the liver, including steatosis, inflammation, cirrhosis, and complications. Autophagy is critical to maintain liver homeostasis, but dysfunction of autophagy has been observed in ALD. Generally, autophagy is considered to protect the liver from alcohol-induced injury and steatosis. In this review, we will summarize novel modulators of autophagy in hepatic metabolism and ALD, including autophagy-mediating non-coding RNAs (ncRNAs), and crosstalk of autophagy machinery and nuclear factors. We will also discuss novel functions of autophagy in hepatocytes and non-parenchymal hepatic cells during the pathogenesis of ALD and other liver diseases.

## 1. Introduction

Over-consumption of alcohol can cause a spectrum of alcohol-related liver disease (ALD) including alcoholic fatty liver disease, alcoholic hepatitis, cirrhosis, and complications [1]. In the Western world, alcohol abuse disorders affect nearly one in every 10 individuals of the general population, which rendered a significantly increased prevalence of alcohol-related liver disease [1]. The contribution of oxidative stress in ALD has been well accepted, and mechanisms include accumulation of acetaldehyde, increase of nicotinamide adenine dinucleotide (NAD)H/NAD^+^ ratio, and/or generation of reactive oxidative species (ROS) [2]. Involvement of oxidative stress in mitochondrial functions and structures during the progress of ALD can lead to altered oxidative phosphorylation, mitochondrial DNA damage, and mitochondrial protein profiles change [3,4,5,6,7,8]. Moreover, lipid peroxidation induced by the accumulation of oxygen free radicals and lipids from alcohol metabolism can further enhance oxidative damage in ALD [9].

Autophagy (from the Greek, “auto” oneself, “phagy” to eat) is a critical degradation process that delivers cytoplasmic cargo (macromolecules or organelles) to the lysosome [10]. Autophagy is evolutionally conserved, and its dysfunction is associated with the pathogenesis of multiple diseases, including tissue injury, microbial infection, cancer, neurodegeneration, and aging [10]. Three major types of autophagy are frequently studied, including macroautophagy, microautophagy, and chaperone-mediated autophagy (CMA) [11]. The macroautophagic process involves the sequestration of cytosolic materials into autophagosomes, transport of autophagosomes to the lysosome, and formation and degradation of autolysosomes [11]. Microautophagy is mainly defined in yeast but also can be observed in mammalian cells. The microautophagic process refers to a direct engulfment of cytoplasmic cargo at the limiting membrane of the lysosome, which mediates both invagination and vesicle scission into the lumen of lysosomes [11]. CMA is mediated by chaperones, e.g., heat shock-cognate protein of 70 kDa (HSC70), and specific protein targets are shuttled via the chaperones across the lysosomal membrane for degradation in the lumen [11]. Among these three types of autophagic process, macroautophagy, referred to hereafter simply as autophagy, is the most active form and has been widely studied in liver physiology and diseases. Details regarding autophagic process and functions of autophagy-related genes have been described in other recent reviews [10,12,13].

Autophagy is affected in ALD. Autophagy can be induced by alcohol and can be selective for damaged mitochondria and accumulated lipid droplets, but not for long-lived proteins [14]. Multiple mechanisms are involved in this induction, including ethanol metabolites (mostly acetaldehyde) [15], ROS [14,16], and alteration of signaling pathways (such as the mammalian target of rapamycin (mTOR), 5′ AMP-activated protein kinase (AMPK), and forkhead box O (FOXO)3a pathways) [17,18,19,20]. Alcohol-induced proteasome inhibition, endoplasmic reticulum (ER) stress, and metal elements like zinc, are also involved in alteration of autophagy following ethanol treatment [21,22,23]. Interestingly, autophagy can be suppressed in chronic ALD. In a mouse model with Lieber-DeCarli diet containing different levels of alcohol for 4 weeks, autophagy is stimulated by a lower dose of alcohol (accounting for 29% of the caloric need), but suppressed by a higher dose (accounting for 36% of the caloric need) [24]. Alcohol-induced liver injury is aggravated by further suppression of autophagy while improved by autophagy activation [24].

An increasing amount of evidence suggests that dysfunction of autophagy is associated with the progress of fatty liver disease. Some of the progresses have been discussed in recent reviews [25,26]. In this review, we will summarize new findings regarding the signaling and roles of the macroautophagic process in ALD, focusing on novel factors that have been shown to mediate autophagy during the pathogenesis of ALD.

## 2. General Interactions between Autophagy and Liver Diseases

Evidence from patients and animal models has suggested that autophagy is altered but plays critical roles in multiple types of liver disease, including virus infection, non-alcoholic fatty liver disease, alcoholic fatty liver disease, drug-induced liver injury, liver fibrosis, and hepatocellular carcinoma [11,13,25,26,27,28]. By taking advantage of liver-specific loss of autophagy-related genes (*Atg*), several new factors have been defined in the pathological changes induced by autophagy deficiency, including High mobility group box 1 (HMGB1) [29], Yes-associated protein (YAP) [30], and mTOR [31]. Herein, we will summarize new findings regarding the interactions between autophagy and liver diseases, focusing on autophagy in hepatic lipid metabolism, interactions between autophagy and ncRNA, and the effects of autophagy in non-parenchymal liver cells on liver diseases.

### 2.1. Autophagy in Hepatic Lipid Metabolism

The liver is critical to the metabolism in the human body [32]. Generally speaking, autophagy can affect hepatic metabolic homeostasis via three approaches, including acceleration of nutrient recycle, removal of abnormal organelles and toxic protein aggregates, and regulation of the levels of metabolic factor [26]. Hepatic steatosis is one major manifestation of liver dysfunction. Lipid droplets (LDs) are the storage form of lipids. Previous studies have revealed an important role of autophagy in the breakdown of LDs [33]. In this section, we will discuss novel players that regulate lipid metabolism by altering autophagy, and the interactions between autophagy and key metabolic factors.

#### 2.1.1. Autophagy as A Key Executor in Hepatic Steatosis

Lipophagy is a type of selective autophagy that targets to LDs [10]. Inhibition of autophagy by either pharmaceutical treatment using 3-methyladenine (an autophagy inhibitor) or RNA interference against *Atg5* or *Atg7* can increase LD number and size in both cultured hepatocytes and mouse livers [33]. In non-alcoholic fatty liver disease (NAFLD), autophagy can be impaired at different levels with changes of multiple signaling pathways and regulators [25,26]. Considering the strong association between autophagy dysregulation and NAFLD, autophagy has become one promising target for the treatment of NAFLD [11].

Glucose-6-phosphatase α (G6PC) functions in the final step of gluconeogenesis and glycogenolysis by catalyzing the formation of free glucose from glucose-6-phosphate [34]. Lack of G6PC can cause glycogen storage diseases Ia (GSDIa) and derangements in hepatic metabolism [34]. Interestingly, autophagy was found to be impaired in G6PC-deficienct cells, and in the murine and canine models of GSDIa [34], which was coupled with stimulation of mTOR and inhibition of AMPK pathways. Activation of autophagy by rapamycin was able to reduce hepatic triglyceride content in GSDIa models [34]. The study provides strong evidence that over-loaded nutrients can suppress autophagy, which is critical to the progression of diseases related to metabolic derangements.

Several factors that regulate lipid metabolism have been shown to affect autophagy. Adipose triglyceride lipase (ATGL), a major hepatic lipase that regulates triglyceride turn-over, was able to promote autophagy [35]. A recently published study shows that knockdown of ATGL in vivo reduces the expression of *Atg* genes, impaired autophagy, and reduced LD localization with microtubule-associated proteins 1A/1B light chain 3 (LC3) [35]. Similar changes were also found in primary mouse hepatocytes following ATGL inhibitor treatment, suggesting that a liver-specific inhibition of ATGL can suppress autophagy/lipophagy [35]. Moreover, ATGL overexpression in the liver could induce autophagy genes’ expression and was sufficient to promote autophagy/lipophagy [35]. Triglyceride turn-over and fatty acid oxidation were both induced by ATGL overexpression in primary mouse hepatocytes, but suppressed by chloroquine, si*Atg5*, and lysosomal acid lipase inhibitor, indicating that lipophagy is required for ATGL-mediated triglyceride catabolism [35]. Finally, effects of ATGL on autophagy/lipophagy were abolished in sirtuin 1 (SIRT1)-deficient livers, suggesting that ATGL-driven autophagy/lipophagy is mediated by SIRT1 [35]. Another study shows that an ER-residential protein, reticulon 4B (NOGO-B), was highly expressed in NAFLD-associated hepatocellular carcinoma (HCC) in both mice and human [36]. NOGO-B expression was induced by CCAAT/enhancer-binding protein beta (CEBPβ), which was upregulated by cluster of differentiation 36 (CD36)-mediated oxidized low-density lipoprotein (oxLDL) uptake [36]. Highly expressed NOGO-B could interact with ATG5 to promote lipophagy, and finally enhanced YAP oncogenic activity [36]. Interestingly, Angelina S. Gross et al. [37] shows that acetyl-CoA carboxylase 1 (ACC1), a rate-limiting enzyme for the de novo synthesis of lipids, was associated with autophagy activation as a downstream factor of AMPK. Although the study only shows evidence from aging yeast, it presents a potential interaction between lipogenesis and autophagy pathways.

In addition to the direct observations of autophagy dysfunction in models of metabolic derangements, one autophagy-related factor was found to be involved in hepatic lipid metabolism. RUBICON is a RUN domain-containing BECLIN1-interacting protein, which can bind to the core class III phosphatidylinositol-3 kinase (PI3KC3)/vacuolar protein sorting 34 (VPS34) complex and inhibit PI3KC3/VPS34 activity [11]. RUBICON therefore can be a strong suppressor of autophagy and thus a valuable therapeutic target [11]. Expression of *Rubicon* was enhanced in HepG2 cells treated with palmitic acid, in mouse livers following high fat diet treatment, and in liver samples from NAFLD patients, all suggesting that an up-regulation of RUBICON was associated with NAFLD [38]. Further studies showed that knockdown of *Rubicon* by siRNA could reduce palmitate-induced lipotoxicity in HepG2 cells. Moreover, hepatic-specific *Rubicon* knockout mice were resistant to HFD-induced hepatic steatosis and injury, suggesting that *Rubicon* may be a suitable therapeutic target for NAFLD [38].

#### 2.1.2. Crosstalk of Autophagy and the Nutrient-Sensing Nuclear Receptors

Regulation of autophagy by nutrient-sensing signaling pathways, like AMPK and mTOR pathways, is well described [12,13]. Recent evidence has also shown that autophagy can interact with certain nuclear receptors and further affect hepatic metabolic changes. Fasting-state and fed-state can lead to activation of different nuclear receptors in the liver. The effect of transcription factor EB (TFEB) on autophagy has been well studied in previous studies [39,40]. Interestingly, two recent articles show that activation of a nuclear receptor, farnesoid X receptor (FXR), strongly suppressed autophagy-related gene expression during fed-state, which led to feeding-mediated suppression of autophagy [41,42]. Conversely, fasting-activated nuclear receptors, peroxisome proliferator-activated receptor alpha (PPARα) [41], and cAMP response element-binding protein (CREB) [42], were found to induce expression of autophagy-related genes and cause fasting-mediated activation of autophagy. Interestingly, another currently published study shows that CREB deficiency could lead to hepatic autophagy suppression and additional lipid accumulation upon starvation [43]. Their further study suggests that CREB interacted with PPARα and PPAR gamma coactivator 1-alpha (PGC1α) to regulate autophagy-related genes expression and nuclear translocation of TFEB [43]. These studies provide strong evidence that different nuclear receptors may coordinate the function of autophagy at the transcriptional level under different nutritional states, and there are interactions among different nuclear receptors in regulating autophagy.

PPARα is activated during fasting and plays an important role in regulating the expression of genes in lipid catabolism [44]. Its activation during fasting-state is involved in fasting-mediated activation of autophagy [41]. Nevertheless, autophagy is also shown to modulate PPARα activity by regulating levels of PPARα repressors. Iershov, A., et al. have studied roles of PI3KC3 in coordinating autophagy and mitochondrial lipid catabolism in the liver [45]. Firstly, they found that deletion of *Vps15*, an important regulatory subunit of PI3KC3, in the liver caused mitochondrial depletion, PPARα inactivation, and fatty acid oxidation defect. Treatment of PPARα agonist, fenofibrate, restored lipid catabolism and rescued mitochondrial function. The transcription factor co-activator PGC1α could also rescue mitochondria activity in *Vps15*-deficient hepatocytes. Mechanically, autophagy could degrade PPARα repressors histone deacetylase 3 (HDAC3) and nuclear receptor corepressor 1 (NCoR1), and inhibition of HDAC3 improved PPARα responses in *Vps15*-deleted livers. They also found that both NCoR1 and HDAC3 bound with GABA type A receptor-associated protein (GABARAP) and LC3 with higher affinities to GABARAP [45]. In other autophagy deficiency mouse models with liver-specific deletion of *Atg5* or *Atg7*, NCoR1 was further identified to bind to the autophagosomal GABARAP family proteins and its turn-over was affected by autophagy, which was responsible for PPARα-inactivation and consequently the retarded lipid oxidation in autophagy-deficient livers [46]. Another study from the same group shows that fasting-induced lipid droplet formation was decreased in *Atg7*-deleted livers, which is possibly associated with the suppressed expression of liver X receptor alpha (LXRα) by NCoR1 [46,47]. It is worth noting that fasting-induced hepatic steatosis was shown to be impaired in liver-specific *Atg5*-deficient mouse liver in another study, in which further deletion of nuclear factor erythroid 2-related factor 2 (NRF2) was able to rescue impaired lipid droplet formation in *Atg5*-deleted livers [48]. However, sequestosome-1 (*Sqstm1*) knockout in *Atg7* or *Atg5*-deleted livers could alleviate NRF2 activation but not affect NCoR1 accumulation and consequently PPARα suppression [46], suggesting that NCoR1 accumulation was rather controlled by autophagy function than NRF2 activation. Further deletion of *NCoR1* generated more severe pathological change in *Atg7*-deleted livers, but not in livers with *Atg7* and *Sqstm1* both deleted [46], indicating a potential interaction between NCoR1 and the SQSTM1/NRF2 pathway, which may explain some of discrepancy in different studies [47,48].

**Figure 1:**
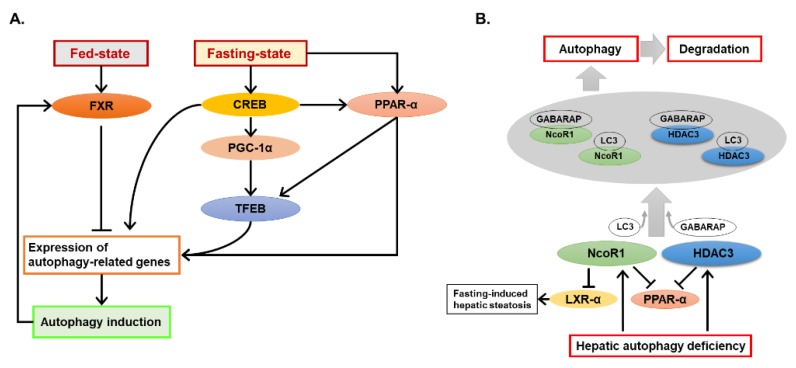
Crosstalk of autophagy and nutrient-sensing nuclear receptors. (**A**) Roles of nutrient-sensing nuclear receptors in autophagy regulation under the fasting and fed states. Feeding-induced farnesoid X receptor (FXR) activation inhibits the expression autophagy-related genes and thus suppresses autophagy. In fasting status, cAMP response element-binding protein (CREB) and peroxisome proliferator-activated receptor alpha (PPARα) are activated. Both of them can induce expression of autophagy-related genes directly or indirectly by activating transcription factor EB (TFEB), and therefore activate autophagy. Conversely, FXR is suppressed in *Atg7*-deleted livers and is associated with *Atg7* deletion-induced cholestasis. Autophagy may thus be important in sustaining the expression and function of FXR. (**B**) Nuclear receptor corepressor 1 (NCoR1) and histone deacetylase 3 (HDAC3) are two PPARα suppressors. Both of them can bind to GABA type A receptor-associated protein (GABARAP) and microtubule-associated proteins 1A/1B light chain 3 (LC3) with a higher affinity to GABARAP, and then be degraded by the autophagy process. In mice with liver-specific loss of *Atg7*, liver X receptor alpha (LXRα) can be inhibited by accumulation of NcoR1, which is associated with decrease of fasting-induced lipid droplet formation in these mice.

Other than PPARα, FXR was also found to be suppressed in autophagy-deficient livers and plays an important role in autophagy deficiency-induced cholestatic injury [49]. All the emerging evidence strongly suggests that interactions between autophagy and nutrient-sensing nuclear receptors are critical to hepatic metabolism homeostasis. Taken together, all studies presented above suggest that autophagy plays a central regulating role in nutrient metabolism. Autophagy not only serves as a degrading mechanism for some key modulators in nutrient catabolism, but can also modulate metabolic signaling. Additionally, the crosstalk with nutrient-sensing transcription factors renders a more powerful effect of autophagy on metabolism by transcriptional regulation. The new findings in crosstalk of autophagy and nutrient-sensing nuclear receptors is summarized in Figure 1.

### 2.2. Regulation of Autophagy by Non-Coding RNAs in the Liver

Protein factors are powerful regulators for the maintenance of normal biological process. However, non-coding RNAs (ncRNA), which used to be called “junk RNA”, have been recently deeply studied with critical biological roles [50]. In this section, we will discuss recently published studies regarding microRNAs (miRNA) and long non-coding RNAs (lncRNA), two widely studied ncRNAs, in hepatic autophagy regulation.

The discovery of miRNAs establishes a novel post-transcriptional regulation of gene expression and these ncRNAs have been involved in most biological pathways [51]. MiRNAs are a class of short ncRNAs (normally containing about 22 nucleotides) that can guide the binding of the RNA-induced silencing complex to target messenger RNA molecules, leading to degradation and/or translational inhibition of messenger RNA [51]. Dozens of miRNAs have been found to regulate autophagy with impacts on the core autophagy pathways [51,52,53]. In the liver, *miR-26* could inhibit autophagy and enhance chemosensitivity of HCC cells [54]. In this study, Jin et al. found a significant decrease of *miR-26a/b* level in HCC cells following treatment of chemotherapeutic drug doxorubicin (Dox) [54]. Further mechanistic study identified an Unc-51 like autophagy activating kinase 1 (*Ulk1*) as a target of *miR-26a/b* [54]. A decrease of *miR-26a/b* level was also observed in tumors from HCC patients, which was negatively correlated with the protein level of ULK1 [54]. Another interesting study designed engineered adipose-derived mesenchymal stem cells to overexpress *miR-181-5p*, and found that exosomes from these cells were able to transfer *miR-181-5p* to mouse hepatic stellate (HST-T6) cells or a carbon tetrachloride (CCL_4_)-induced liver fibrosis mouse model, leading to inhibition of fibrosis pathway and CCL_4_-induced liver fibrosis by autophagy activation [55].

LncRNAs are another set of important ncRNAs, which have a length exceeding 200 nucleotides but are not able to translate into proteins [56]. Expressions of both autophagy-inducing and -inhibiting lncRNAs can be altered in cancers [56]. Expressions of the autophagy-inducing lncRNAs, including HNF1A antisense RNA 1 (*Hnf1a-as1*), HOX transcript antisense RNA (*Hotair*), and hepatocellular carcinoma up-regulated long non-coding RNA (*Hulc*), were up-regulated in HCC [56]. Another autophagy-inducing lncRNA, phosphatase and tensin homolog pseudogene 1 (*Ptenp1*), was down-regulated in HCC [56]. Mechanically, *Hnf1a-as1* could promote autophagy by sponging *miR-30b-5p*, and functioned as an oncogene [57]. Similarly, *Ptenp1* was able to induce autophagy by decoying onco-miRs *miR-17*, *miR-19b*, and *miR-20a*, which otherwise would target autophagy-related genes, including *Ulk1*, *Atg7*, and *Sqstm1*, and the negative regulators of protein kinase B (AKT) pathway, including phosphatase and tensin homolog (*Pten*) and PH domain and leucine rich repeat protein phosphatase (*Phlpp*) [58]. Abnormal expression of *Hotair* has been found in different types of cancers. In HCC its overexpression was associated with tumor size and it could activate autophagy by upregulating *Atg3* and *Atg7* in HCC [59]. Another interesting lncRNA, *Hulc*, was originally characterized to be highly up-regulated in HCC, and was dysregulated in several other cancers as well [56,60]. In HCC, *Hulc* overexpression could stimulate autophagy and attenuates the chemosensitivity of several chemo drugs in HCC cells [61]. Mechanically, *Hulc* may trigger autophagy by stabilization of SIRT1 [61]. Finally, a small ncRNA, Vault RNA1-1 (*vtRNA1-1*), could bind with SQSTM1 and interfere with SQSTM1 multimerization, which led to riboregulation of SQSTM1-dependent autophagy and aggregate clearance [62]. Overall, these studies present strong evidence that ncRNAs have impacts on autophagy by regulating different autophagy pathways.

### 2.3. Effects of Autophagy in Non-Parenchymal Hepatic Cells on Liver Diseases

Parenchymal hepatocytes occupy about 70–85% of the liver volume and are responsible for the major liver functions [63]. As such, most studies regarding the functions of autophagy in the liver focus on autophagy in parenchymal hepatocytes. Non-parenchymal cells constitute only 6.5% of liver volume, they nevertheless contribute 40% to the total number of liver cells [63]. At least three types of non-parenchymal cells are localized in hepatic sinusoidal compartments, including endothelial cells, Kupffer cells (KC), and hepatic stellate cells (HSC) [63,64]. Unlike autophagy in parenchymal hepatocytes, autophagy in the non-parenchymal cells of the liver has not been fully studied. Generally, autophagy in KCs and endothelial cells is critical to maintain the homeostasis of these cells. However, in HSCs, autophagy is required for their activation [64].

Autophagy dysfunction of non-parenchymal cells in the liver can affect the pathogenesis of liver diseases, particularly the progress of liver fibrosis. Autophagy in HSCs can be induced by several factors, including hypoxia-inducible factor-1α (HIF-1α), transforming growth factor β1 (TGF-β1), and HMGB1 [64]. Autophagy was up-regulated in HSCs after liver injury in mice, and inhibition of autophagy could decrease fibrogenesis of JS1 cells, an immortalized mouse HSC line [65]. Specific deletion of *Atg7* in mouse HSCs could attenuate chronic injury-induced liver fibrosis, which was associated with autophagy-mediated lipid metabolism [65]. This study suggests activation of HSCs requires production of energy from autophagy-mediated lipid catabolism. There is another study showing that autophagy was involved in the activation of HSCs by ER stress [66], further supporting the critical role of HSC autophagy in the progress of liver fibrogenesis. In contrast to the consequence of autophagy activation in HSCs, autophagy in macrophages and endothelial cells could protect the liver from fibrogenesis. Macrophage-specific deletion of *Atg5* could aggravate CCL_4_-induced liver fibrosis [67]. Higher levels of interleukin (IL)-1α and IL1β and further recruitment of inflammatory cells were also observed in mouse livers with *Atg5* deletion in macrophages [67]. Impaired autophagy by deletion of *Atg7* in endothelial cells did not affect liver homeostasis but amplified liver fibrosis without increasing liver injury following CCL_4_-treatment [68].

In addition to impacts on liver fibrosis, impaired macrophage autophagy by *Atg5* deletion could promote proinflammatory macrophage polarization and enhance the immune response in obese mice [69]. Macrophage autophagy inhibition could also increase toxin-induced acute liver injury from D-galactosamine/lipopolysaccharide (GalN/LPS) through down regulation of IL1β [70]. These studies uncovered critical roles of autophagy of non-parenchymal cells in the pathogenesis of liver diseases, which were underappreciated. Roles of autophagy in different hepatic cells in the pathogenesis of liver diseases are summarized in Table 1.

## 3. Alcohol Modulates Autophagy in the Liver via Multiple Pathways

ALD exhibits several pathological features, including steatosis, inflammation, fibrosis, and cirrhosis [1]. Recently, changes of several novel autophagy modulators have been associated with the pathogenesis of ALD, which are discussed here.

### 3.1. Novel Autophagy-Mediating Molecules in ALD

#### 3.1.1. Novel Modulators of Alcohol-Induced Lipophagy 

It is well established that alcohol can disturb hepatic lipid homeostasis via multiple mechanisms, including activation of hepatic lipid synthesis, excessive uptake of fatty acids by the liver, and lower secretion of lipoproteins [77]. Retarded lipolysis also occurs in the liver following alcohol consumption, evidenced by a lower activation of lipases, ATGL and hormone-sensitive lipase (HSL), by β-Adrenergic signaling [78], and impairment of mitochondrial oxidation of fatty acids [79].

Lipophagy is a selective form of autophagy mediating lipid droplets degradation in lysosomes [77]. Emerging evidence shows that lipophagy plays a key role in lipid catabolism, and its blockage in ALD contributes to alcohol-induced liver steatosis. Our recent study shows that alcohol treatment elevated lipid content in an immortalized mouse hepatocyte line, which could be enhanced by pharmacological inhibition of autophagy or knockdown of either *Sqstm1* or *Atg5* [80]. Mechanically, SQSTM1 potentially bridged autophagosomes and lipid droplets by binding with ubiquitinated proteins on lipid droplets [80].

In addition to the involvement of the conventional autophagic pathway, evidence shows that several novel factors are associated with impairment of lipophagy induced by alcohol. Dynamin2 (DYN2) is a large GTPase involved in membrane deformation and cellular protein trafficking [81]. An early study shows that DYN2 was required for lipid droplet breakdown in hepatocytes by mediating autophagic lysosomal reformation and lysosomal tubule scission [81]. Interestingly, DYN2 activity was reduced in hepatocytes from alcohol-fed rats, suggesting a potential association of DYN2 suppression with inhibited lipophagy from alcohol treatment [82]. Another GTPase, RAB7, which is known for its involvement in the late endosome pathway, is also associated with the dynamic of LDs [83]. In their study, Barbara Schroeder et al. show that RAB7 was essential to LD breakdown by recruiting degradative compartments and was indispensable for the proper execution of lipophagy [83]. Another recent study from the same group shows that RAB7 activity was markedly decreased in hepatocytes from alcohol-fed rats, suggesting that RAB7 inhibition by alcohol may account for alcohol-induced lipophagy inhibition and hepatic steatosis [84]. Taken together, these studies suggest that molecules important in the membrane trafficking pathway can also be critical to hepatocellular lipophagy and that alcohol-induced hepatic steatosis may be partially associated with the dysfunction of these factors. However, experiments in these studies were mostly performed in vitro. Evidence from alcohol-fed animals are still required to clarify roles of membrane trafficking factors in alcohol-induced lipophagy inhibition.

#### 3.1.2. TFEB in ALD

Chronic alcohol treatment can decrease both the amount and the function of lysosomes in rat livers, which may contribute to autophagy suppression [85,86,87]. However, the exact mechanism of lysosome dysfunction following alcohol treatment is still unclear. *Tfeb* is a master gene for lysosomal biogenesis, which can also regulate starvation-induced autophagy by controlling expression of autophagy-related genes [39,40]. Acute alcohol administration increased nuclear levels of TFEB in mouse hepatocytes, while decreased nuclear levels of TFEB is observed in mouse hepatocytes following chronic alcohol administration [88]. This is the first study that describes that alcohol alters levels of nuclear TFEB in the liver, which can modulate hepatic autophagy differentially following acute alcohol gavage and chronic alcohol feeding. However, the causal role and mechanisms by which TFEB affects ALD progress were still unclear until a recent study was published. In this study, Xiaojuan Chao et al. elucidated the functions of TFEB in alcohol-induced liver steatosis and injury in animal models [72]. TFEB proteins were decreased in both total lysates and nuclear fractions of the mouse liver following a chronic-plus-binge and a long-term chronic alcohol treatment, but not in either a short-term chronic or an acute gavage alcohol treatment. Decrease of nuclear TFEB proteins was further confirmed in human alcoholic hepatitis livers, indicating their finding may be clinically relevant. In order to demonstrate the function of TFEB in ALD, the authors first knocked down hepatic *Tfeb* by shRNA in mice and found that TFEB knockdown exacerbated liver injury and steatosis induced by chronic-plus-binge alcohol treatment. Furthermore, enhanced liver injury by alcohol treatment was also observed in *Tfeb*/transcription factor E3 (*Tfe3*) double-knockout mice. Finally, overexpression of TFEB in the liver compromised alcohol-induced liver injury. With these results, the authors have convincingly shown a strong connection between TFEB and alcohol-induced liver injury.

The mTOR is a kinase that receives signals from growth factors and nutrients, and plays critical roles in regulating essential cellular functions related to the promotion of cell growth and metabolism [89]. Phosphorylation of TFEB by mTOR can decrease TFEB activity and promote its proteasomal degradation [90]. The authors thus determined mTOR activation in their model and found that the activity of mTOR was induced by chronic-plus-binge alcohol treatment with significantly increased phosphorylation levels of ribosomal protein S6 (S6) and eukaryotic translation initiation factor 4E-binding protein 1 (4E-BP1), two classical mTOR downstream factors [72]. Pharmacologic inhibition of mTOR by torin-1, a novel mTOR inhibitor, could reverse the inhibition of TFEB, liver steatosis, and liver injury in chronic-plus-binge alcohol treatment, suggesting a potential association between the mTOR pathway and TFEB inhibition by alcohol [72]. Mechanical studies indicated that inhibition of TFEB could affect lysosomal biogenesis and mitochondrial functions, which would be related to alcohol-induced steatosis and liver injury [72]. This study extends the knowledge of the regulatory function of autophagy in ALD, and provides a potential therapeutic target, TFEB, for ALD.

Interestingly, mTOR-mediated autophagy regulation was also found in another transgenic mouse model. Aldehyde dedydrogenase-2 (ALDH2) is a critical enzyme for acetaldehyde metabolism [91]. In *Aldh2* transgenic mice, ethanol-induced hepatic steatosis and inflammation were improved compared to control mice, which was associated with the maintenance of autophagy through the AKT-mTORC1-ULK1 pathway [91]. Other than interaction with the autophagy pathway, the mTOR pathway also contributed to alcohol-induced liver steatosis and injury by regulating lipogenesis [92]. Hyperactivation of mTORC1 was observed in livers of alcohol-fed mice and patients with ALD, which was likely associated with defects of the DEP domain-containing mTOR-interacting protein (DEPTOR) and the nicotinamide adenine dinucleotide-dependent deacetylase SIRT1 in the liver [92]. Overexpression of DEPTOR ameliorated, whereas hepatocyte-specific deletion of SIRT1 exacerbated, alcoholic steatosis, inflammation, and liver injury in mice [92]. Pharmacological intervention with rapamycin, an mTOR inhibitor, could also improve alcohol-induced steatogenic phenotypes [92]. These two studies strengthened the hypothesis that mTOR pathway is a critical determinant of ALD pathogenesis, which can be due to its functions in regulating autophagy and lipogenesis.

#### 3.1.3. Potential Involvement of Chaperone-Mediated Autophagy in ALD

As macroautophagy is the most active form of autophagic process, most autophagy-related studies in ALD investigate the involvement of macroautophagy. Interestingly, emerging evidence now indicates CMA also regulates alcohol-induced liver injury and steatosis. Sorting nexin (SNX)-10 has been reported to regulate endolysosomal trafficking and stabilization, which can contribute to the CMA process [93]. The role of SNX10 in ALD has been examined in *Snx10* knockout mice given Lieber-DeCarli alcohol diet and in primary *Snx10* knockout hepatocytes cultured with alcohol. Yan You et al. found that *Snx10* knockout mice were resistant to alcohol-induced liver injury and steatosis [93]. Upregulation of lysosome-associated membrane protein 2A (LAMP2A) was observed in *Snx10*-deficient livers, and interference of LAMP2A suppressed the activation of NRF2 and AMPK signaling that was induced by *Snx10* deficiency. It seems that SNX10 induced NRF2 and AMPK activation relies on CMA activation [93]. Mechanically, SNX10 could bind with cathepsin A (CTSA), an enzyme that assists LAMP2A degradation. Deficiency in *Snx10* inhibited CTSA maturation, and thus increased LAMP2A stability [93]. This study demonstrates that deficiency of a CMA negative regulator, SNX10, can protect mice from alcohol-induced liver injury and steatosis, providing evidence for SNX10 as a potential therapeutic target for ALD.

Lipocaline-2 (LCN2) deficiency may also maintain hepatic CMA activity in mouse livers following chronic alcohol treatment [94]. Together with the SNX10 study, it seems that impaired CMA may contribute to alcohol-induced liver injury and hepatic steatosis.

### 3.2. Autophagy-Mediating miRNAs in ALD

As discussed in previous sections, emerging evidence has indicated the involvement of miRNAs in autophagy regulation. In ALD, several miRNAs have been reported to affect alcohol-induced liver injury and steatosis by altering autophagy. Different from the study in HCC cells [54], Fangfang Jin et al. [95] showed that *miR-26a* could protect against alcohol-induced acute liver injury by targeting dual specificity protein phosphatase (DUSP)-4 and DUSP5, two MAPKs inhibitors. Activation of MAPKs by *miR-26a* then induced *Beclin-1* expression and thus activated autophagy. This study suggests that autophagy-mediating miRNAs can be involved in ALD.

**Figure 2:**
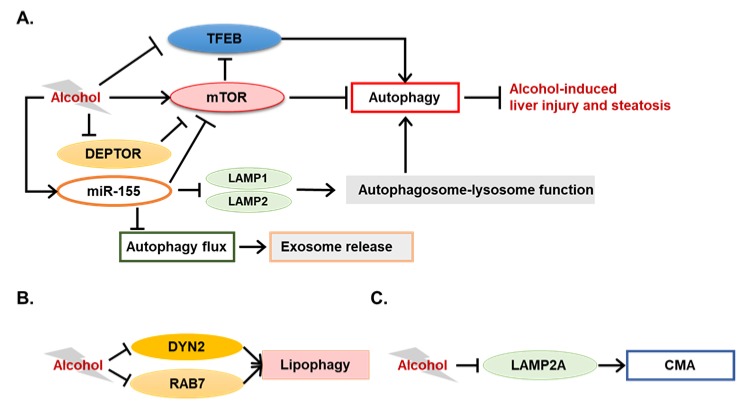
Novel modulators of autophagy in alcohol-related liver disease (ALD). (**A**) Alcohol can suppress transcription factor EB (TFEB) and inhibit autophagy by affecting lysosomal biogenesis. This suppression of TFEB is associated with alcohol-mediated activation of the mammalian target of rapamycin (mTOR). As a strong inhibitor of autophagy, mTOR has been shown as a central factor bridging autophagy and ALD. An mTOR inhibitor, DEP domain-containing mTOR-interacting protein (DEPTOR), is suppressed by alcohol, which is associated with the pathogenesis in ALD. Interestingly, an alcohol-induced miRNA, *miR-155*, can inhibit mTOR and induce expression of genes that can initiate autophagy, while repressing the expression of lysosome-associated membrane protein (*Lamp*)-1 and *Lamp2*. The overall effects of *miR-155* are to impair autophagy and to contribute to alcohol-induced liver steatosis, inflammation, and exosome release. What should be noted is that the discrepancy regarding mTOR activity exists among results from different laboratories, suggesting that mTOR activity may be varied by factors other than alcohol use. (**B**) Alcohol-treatment can decrease Dynamin2 (DYN2) and RAB7 expression in rat livers, both of which are critical to lipophagy. (**C**) Alcohol can suppress LAMP2A expression, which is a key factor during the process of chaperone-mediated autophagy (CMA).

Another study shows an interesting link between miRNA-mediated autophagy dysregulation and ALD. In this study, Mrigya Babuta et al. provide evidence for *miR-155* as a mediator of alcohol-related exosome production and autophagy dysfunction in hepatocytes and macrophages [96]. Expression of *miR-155* was induced by alcohol but deletion of *miR-155* protected mice from alcohol-induced steatosis and inflammation [96]. Both autophagy and mTOR pathways were impaired in ALD patient livers and in mouse livers following either a 5-week chronic alcohol treatment or a chronic-plus-binge alcohol treatment [96]. Interestingly, *miR-155* deficiency could protect mice from alcohol-induced decrease of mTOR and ras homolog enriched in brain (RHEB) and accumulation of SQSTM1 and LC3-II protein, whereas overexpression of *miR-155* reduced the protein levels of mTOR and RHEB in both hepatocytes and macrophages [96]. These results suggest that *miR-155* contributed to alcohol-induced dysfunction of autophagy and mTOR pathway. Further experimental analysis showed that *miR-155* likely suppressed autophagy by targeting to *Lamp1* and *Lamp2*, which may contribute to defects in the autophagosome-lysosome fusion step and thus suppressed autophagic substrates degradation [96]. Another interesting finding in this study is the regulation of exosome release by *miR-155*. Serum levels of exosome were increased in ALD patients and alcohol-fed mice, whereas *miR-155* deficiency significantly limited exosome release even in pair-fed mice [96]. Exosome release by alcohol-treatment was confirmed in both primary hepatocytes and Kupffer’s cells, which was suppressed in *miR-155* deficiency. The authors also found an increase of *miR-155* level in circulating exosomes following alcohol treatment. Surprisingly, either pharmacological inhibition of lysosomes or knockdown of LAMP1 and LAMP2 increased exosome release in macrophages and hepatocytes [96]. Taken together, this study comprehensively delineates a crosstalk among miRNA, autophagy, and exosome production in ALD, suggesting an atypical role of autophagy as a promoter of exosome production. As several key experiments in this study were performed using in vitro models, functional studies using ALD mouse models are necessary for further understanding the roles of miRNA-autophagy-exosome in ALD pathology. A summative figure of new modulators of autophagy and their functions in ALD is shown in Figure 2.

## 4. Roles of Autophagy during Pathogenesis of ALD

In this section, we will summarize recently reported studies regarding roles of autophagy modulation in ALD. As discussed above in the previous section, autophagy in different hepatic cells may have different functional consequences in liver diseases. Here, we will discuss emerging evidence of the variable relationships between autophagy and ALD based on the types of cells in the liver, including parenchymal hepatocytes, macrophage, and HSCs (Table 1).

### 4.1. Hepatocytes

Consistent with earlier findings that autophagy can protect animals from alcohol-induced liver injury and steatosis, several recent studies confirmed the ameliorative effects of hepatic autophagy in multiple ALD animal models, including an 8-week alcohol-fed rat model [97] and an alcohol-plus-LPS ALD mouse model [98]. Augmenter of liver regeneration (ALR) is a factor that can promote liver growth and it has been shown to protect against acute liver injury from variable insults [99]. In a recently published study, Limin Liu et al. reported that hepatic ALR could protect mice from alcohol-induced live injury by using an acute mouse model [99]. Mechanically, ALR could activate autophagy, likely by inhibiting the mTOR pathway [99].

Autophagy plays a critical role in maintaining normal liver functions, whereas it can be affected by multiple factors, including alcohol, Western diets, and viral infection [13]. Since ALD often occurs in the presence of pathologic insults other than alcohol, different autophagy functional status may be further modulated leading to diverse consequences in ALD. Recently, we showed that the relationship between autophagy and ALD can be affected by multiple factors, including the particular autophagy genes compromised, the timing and the severity of autophagy dysfunction, and the way alcohol is given [71]. In this study, we examined mouse lines with liver-specific deletion of *Atg5* or *Atg7* and applied two different alcohol models, including an acute model and a chronic-plus-binge model. Acute alcohol binge exacerbated injury and steatosis in *Atg5*-deleted but not Atg7-deleted livers. Similar to a previous study by using siRNA knockdown *Atg7*, mice with inducible deletion of *Atg7* in the liver are more susceptible to alcohol-induced liver injury and steatosis [14]. These results are consistent with the notion that autophagy can protect mice from liver injury and steatosis induced by acute alcohol treatments. In order to further examine the impact of hepatic autophagy dysfunction in ALD, these mouse lines were given alcohol diet in a recently established chronic-plus-binge scheme [71,100]. The results were as expected in that either acute deletion or constitutive deletion of hepatic *Atg7* enhanced alcohol-induced liver injury. Interestingly, chronic-plus-binge alcohol treatment only slightly increased hepatomegaly and induced steatosis in mice with constitutive deletion of hepatic *Atg5*. However, unexpectedly, liver injury caused by *Atg5* deletion was reduced following alcohol treatment in this scheme [71]. Further evidence showed that chronic-plus-binge alcohol treatment reduced Kupffer’s cell infiltration, fibrosis, and cholestatic injury in the *Atg5*-deficient livers. Finally, we examined the expression of genes related to alcohol metabolism, and the results showed that the mRNA levels of cytochrome P450 family 2 subfamily E member 1 (*Cyp2e1*), catalase (*Cat*), several alcohol dehydrogenase (*Adh*) genes, and several *Aldh* genes were decreased in *Atg5*-deficient livers [71]. The protein level of CYP2E1 was also decreased in *Atg5*-deficient livers. In addition, unexpectedly, the plasma alcohol clearance rate was faster in hepatic *Atg5*-deficient mice, suggesting the presence of alternative alcohol-clearance mechanisms in these mice [71]. Overall, this study demonstrates that different autophagy functional status can lead to different consequences from alcohol treatment. It also provides evidence that autophagy may be involved in the regulation of enzyme expression that alcohol metabolism is related to.

### 4.2. Macrophages

As discussed in previous sections, emerging evidence has now shown that autophagy in non-parenchymal liver cells has significant impacts on the progression of liver diseases. Indeed, several studies have shown effects of macrophage autophagy on alcohol-induced liver injury by investigating the myeloid cell-specific *Atg5* or *Atg7* knockout (*Atg5*^*Δmye*^ or *Atg7^Δmye^*) mice. Timothé Denaës et al. found that macrophage autophagy contributed to the protective role of cannabinoid CB2 receptors in ALD, and this protective effect was abrogated by *Atg5*-deletion in macrophages [73]. In this study, *Atg5^Δmye^* mice had comparable levels of liver steatosis and injury as wildtype mice following a short-term chronic-plus-binge alcohol treatment [73]. In a recently reported study, Ghulam Ilyas et al. observed an equivalent level of steatosis but increased mortality in *Atg5^Δmye^* mice following a 21-day chronic alcohol treatment (21 days) plus LPS injection [74], suggesting a protective role of macrophage autophagy in alcohol-induced liver injury. This protective role was confirmed by another group using an *Atg7^Δmye^* mice, which were given a 6-week chronic alcohol treatment [75]. In this study, Shuang Liang et al. found that chronic alcohol feeding plus LPS injection enhanced liver injury in wildtype mice, which was augmented in *Atg7^Δmye^* mice [75]. Both of these studies found an activation of the inflammasome and an elevated IL-1β production in alcohol/LPS-treated *Atg5^Δmye^* and *Atg7^Δmye^* mice [74,75]. Defects in interferon regulatory factor1 (IRF1) degradation and in the removal of damaged mitochondria were associated with enhanced liver injury in *Atg7^Δmye^* mice [75]. Taken together, these studies provide strong evidence that autophagy in macrophages is critical to protect the liver from alcohol-induced damage, either by mediating effects from other protectors or by directly protecting liver from a “second hit” in the progression of liver injury.

### 4.3. Hepatic Stellate Cells

Unlike autophagy in hepatocytes and macrophages, the relationship between autophagy function in HSCs and ALD still lacks evidence. An in vitro study using an immortalized rat HSC line HSC-T6 showed that autophagy could contribute to alcohol-induced HSC activation [76]. Any studies showing roles of HSC autophagy in ALD in an animal model have not been reported. Although fibrosis commonly occurs in human acute alcoholic hepatitis, it is not induced significantly by alcohol in current murine models, except that the treatment combines a high-fat diet with a continuous alcohol treatment [1]. Due to the lack of ALD models for alcohol-induced fibrosis, studying the role of HSC autophagy could be difficult in the scenario of ALD. Generating a suitable model will be beneficial to reveal any possible involvement in the process.

## 5. Conclusions and Perspectives

Current studies have confirmed that autophagy is critical to normal liver functions, and dysfunction of autophagy contributes to the pathogenesis of various liver diseases, including ALD. Generally speaking, research areas in hepatic autophagy have extended from its classic functions of removing abnormal organelles and/or biomolecules to functions of mediating changes of biological pathways. Modulators in multiple metabolic pathways can interact with the autophagy process and their interactions are critical to maintain the homeostasis of liver functions. Novel factors such as ncRNAs have been shown to regulate autophagy in the liver. Moreover, accumulating evidence suggests that important, while underappreciated functions of autophagy in non-parenchymal liver cells, can have significant effects on liver diseases, which can be varied from one cell type to another cell type.

In ALD, the protective roles of autophagy are well established and confirmed by additional evidence from studies of autophagy in different hepatic cells. Several new players have been found in alcohol-mediated regulation of hepatic autophagy, such as TFEB and miRNAs, and regulation of mTOR activity seems to be involved in various alcohol-mediated autophagy changes. Interestingly, CMA is found to be altered by alcohol in the liver. Other types of autophagy may also contribute to the pathogenesis of ALD although the exact functions remain to be revealed.

Taken together, dysfunction of autophagy is strongly associated with the development of liver diseases, indicating a promising strategy for treatment of liver diseases by targeting autophagy. Whereas autophagy in different liver cells can have totally different effects in the pathogenesis of liver diseases, more extensive studies are still required to further understand biological functions of autophagy in non-parenchymal liver cells. In ALD pathogenesis, the role of autophagy in non-parenchymal hepatic cells, especially in HSCs and in endothelial cells, remains elusive. ALD models with significant liver fibrosis would be critical for these studies.

## Figures and Tables

**Table 1:** Autophagy in different types of liver cells.

Cell Types	General Functions	Functions in ALD
Hepatocytes	Autophagy in hepatocytes is critical to maintain homeostasis of liver functions. See other reviews for details [11,13,25,26,27,28].	Generally, autophagy plays a protective role in ALD, whereas different status of autophagy deficiency can lead to diverse consequences [9,14,24,25,26,71,72].
Macrophages	Macrophage autophagy is important to maintain a normal immune response [64]. Macrophage-specific deletion of *Atg5* can aggravate CCL_4_-induced liver fibrosis [67]. Impaired macrophage autophagy can promote proinflammatory macrophage polarization and increase the immune response in obese mice [69]. Inhibition of macrophage autophagy can also increase toxin-induced acute liver injury from GalN/LPS co-treatment through down-regulation of IL1β [70].	Autophagy in macrophages is critical to protect the liver from alcohol-induced damage, either by mediating effects from other protectors or by directly protecting the liver from a “second hit” in the progression of liver injury [73,74,75].
Hepatic stellate cells	Activation of HSCs requires production of energy from autophagy-mediated lipid catabolism [64,65,66].	An in vitro study using HSC-T6, an immortalized rat HSC line, shows that autophagy may contribute to alcohol-induced HSC activation [76].
Endothelial cells	Autophagy in endothelial cells is critical to maintain these cells homeostasis [64]. Impaired autophagy by deletion of *Atg7* in endothelial cells does not affect liver homeostasis but amplifies liver fibrosis without increasing liver injury following CCL_4_-treatment [68].	Unclear.

## Abbreviations

4E-BP1	Eukaryotic translation initiation factor 4E-binding protein 1
ACC1	Acetyl-CoA carboxylase 1
ADH	Alcohol dehydrogenase
AKT	Protein kinase B
ALD	Alcohol-related liver disease
ALDH2	Aldehyde dedydrogenase-2
ALR	Augmenter of liver regeneration
AMPK	5′ AMP-activated protein kinase
ATGL	Adipose triglyceride lipase
CAT	Catalase
CCL4	Carbon tetrachloride
CD36	Cluster of differentiation 36
CEBPβ	CCAAT/enhancer-binding protein beta
CMA	Chaperone-mediated autophagy
CREB	cAMP response element-binding protein
CTSA	Cathepsin A
Cyp2E1	Cytochrome P450 family 2 subfamily E member 1
DEPTOR	DEP domain-containing mTOR-interacting protein
DYN2	Dynamin2
ER	Endoplasmic reticulum
FXR	Farnesoid X receptor
G6PC	Glucose-6-phosphatase α
GABARAP	GABA type A receptor-associated protein
GalN	D-galactosamine
GSDIa	Glycogen storage diseases Ia
HCC	Hepatocellular carcinoma
HDAC3	Histone deacetylase 3
HMGB1	High mobility group box 1
HNF1A-AS1	HNF1A antisense RNA 1
HOTAIR	HOX transcript antisense RNA
HSC	Hepatic stellate cells
HSC70	Heat shock-cognate protein of 70 kDa
HSL	Hormone-sensitive lipase
HULC	Hepatocellular carcinoma up-regulated long non-coding RNA
IL	Interleukin
IRF1	Interferon regulatory factor1
KC	Kupffer cells
LAMP	Lysosome-associated membrane protein
LC3	Microtubule-associated proteins 1A/1B light chain 3
LDs	Lipid droplets
lncRNA	Long non-coding RNAs
LPS	Lipopolysaccharide
LXRα	Liver X receptor alpha
miRNA	MicroRNAs
Mtor	The mammalian target of rapamycin
NAD	Nicotinamide adenine dinucleotide
NAFLD	Non-alcoholic fatty liver disease
NCoR1	Nuclear receptor corepressor 1
ncRNAs	Non-coding RNAs
Nogo-B	Reticulon 4B
NRF2	Nuclear factor erythroid 2-related factor 2
oxLDL	Oxidized low-density lipoprotein
PGC-1α	Peroxisome proliferator-activated receptor gamma coactivator 1-alpha
PHLPP	PH domain and leucine rich repeat protein phosphatase
PI3KC3	Class III phosphatidylinositol-3 kinase
PPARα	Peroxisome proliferator-activated receptor alpha
PTEN	Phosphatase and tensin homolog
PTENP1	Phosphatase and tensin homolog pseudogene 1
RHEB	Ras homolog enriched in brain
ROS	Reactive oxidative species
S6	Ribosomal protein S6
SIRT1	Sirtuin 1
SNX	Sorting nexin
SQSTM1	Sequestosome-1
TFE3	Transcription factor E3
TFEB	Transcription factor EB
Vps34	Vacuolar protein sorting 34
vtRNA1-1	Vault RNA1-1
YAP	Yes-associated protein
